# LASS2 enhances p53 protein stability and nuclear import to suppress liver cancer progression through interaction with MDM2/MDMX

**DOI:** 10.1038/s41420-023-01709-2

**Published:** 2023-11-14

**Authors:** Qingqing Zhao, Wei He, Zhouheng Liu, Liangliang Huang, Xiaoli Yang, Yong Liu, Rui Chen, Xun Min, Yan Yang

**Affiliations:** 1 Department of General Surgery, Affiliated Hospital of Zunyi Medical University, Zunyi, Guizhou China; 2 Department of Laboratory Medicine, Affiliated Hospital of Zunyi Medical University, Zunyi, Guizhou China; 3 School of Laboratory Medicine, Zunyi Medical University, Zunyi, Guizhou China; 4 Department of General Surgery, Shanghai Pudong Hospital, Fudan University Pudong Medical Center, Pudong, Shanghai, China; 5 School of Forensic Medicine, Zunyi Medical University, Zunyi, Guizhou China; 6 Center of Forensic Expertise, Affiliated hospital of Zunyi Medical University, Zunyi, Guizhou China

**Keywords:** Tumour-suppressor proteins, Metastasis, Epithelial-mesenchymal transition, Apoptosis

## Abstract

LASS2 functions as a tumor suppressor in hepatocellular carcinoma (HCC), the most common type of primary liver cancer, but the underlying mechanism of its action remains largely unknown. Moreover, details on its role and the downstream mechanisms in Cholangiocarcinoma (CCA) and hepatoblastoma (HB), are rarely reported. Herein, LASS2 overexpression was found to significantly inhibit proliferation, migration, invasion and induce apoptosis in hepatoma cells with wild-type (HB cell line HepG2) and mutated p53 (HCC cell line HCCLM3 and CCA cell line HuCCT1). Gene set enrichment analysis determined the enrichment of the differentially expressed genes caused by LASS2 in the p53 signaling pathway. Moreover, the low expression of LASS2 in HCC and CCA tumor tissues was correlated with the advanced tumor-node-metastasis (TNM) stage, and the protein expression of LASS2 positively correlated with acetylated p53 (Lys373) protein levels. At least to some extent, LASS2 exerts its tumor-suppressive effects in a p53-dependent manner, in which LASS2 interacts with MDM2/MDMX and causes dual inhibition to disrupt p53 degradation by MDM2/MDMX. In addition, LASS2 induces p53 phosphorylation at ser15 and acetylation at lys373 to promote translocation from cytoplasm to nucleus. These findings provide new insights into the LASS2-induced tumor suppression mechanism in liver cancer and suggest LASS2 could serve as a potential therapeutic target for liver cancer.

## Introduction

Liver cancer, the most common malignant tumor in the world, ranks sixth in primary cancer incidence and the fourth in cancer-related mortality [1]. In recent decades, the incidence rates of liver cancer have increased in several countries [2–4], with over half of the new cases and deaths occurring in China [5]. Owing to varying risk factors, genetic susceptibility, histopathological typing, and tumor microenvironment, the heterogeneity of liver cancer considerably limits its early detection [5]. With the rapid progression of the disease, patients are often diagnosed with advanced stages of liver cancer, thus resulting in an extremely low survival rate. Moreover, owing to the lack of drugs that specifically target the HCC cells, the prognosis is also poor. This highlights the urgency to develop novel therapeutic interventions. The development and progression of liver cancer are associated with tumor suppressor gene inactivation and oncogene activation. An in-depth understanding of these complex cellular and molecular networks will provide new perspectives to improve the outcome of patients with liver cancer.

LAG1 longevity assurance homolog 2 (LASS2), one of the housekeeping genes of the human genome [6–8], is also known as ceramide synthase 2 (CerS2) or tumor metastasis suppressor gene 1 (TMSG1). It is highly expressed in the liver [8, 9]and kidney [8, 10], particularly in hepatocytes [11]. Depending on the cancer type, the heterogeneous expressions of LASS2 are associated with either cancer progression or suppression. Most studies have indicated an interrelationship between low LASS2 mRNA or protein expression and the degree of poor prognosis and invasion in diseases including in liver cancer [12, 13], prostate cancer [14, 15], bladder cancer [16], breast cancer [17, 18]. Thus, LASS2 has been recognized as a tumor suppressor gene. However, a few studies have found that the increased mRNA or protein levels of LASS2 were observed in metastatic cell lines or tumor specimens as compared to non-metastatic cell lines or paracancerous tissue [19, 20]. These increased mRNA or protein levels of LASS2 are correlated with FIGO staging in ovarian cancer patients [20], implicating that LASS2 may act as an oncogene.

In a previous study in HepG2 hepatoblastoma cells (HB) [21], the overexpression of LASS2 inhibited proliferation and induce apoptosis. Studies have reported that low LASS2 expression is associated with a poor prognosis for patients with hepatocellular carcinoma (HCC) [22] and suggested that the combination of LASS2 and TGF-β1 [22] or ASGR1 [23] may assist in predicting the prognosis. All these findings indicate that LASS2 might be independently used as a prognostic marker for HCC patients [23]. To date, the underlying molecular mechanisms of LASS2 reported for HCC regulation remain poorly understood, and its role and mechanisms in other subtypes of liver cancer (such as CCA and HB) are also poorly reported. Therefore, substantial research is necessary before the exact molecular mechanisms of LASS2 tumor-suppressive action is elucidated, and the research in this field needs to be expanded.

p53, a well-known tumor suppressor gene involved in cell cycle control, apoptosis, cell differentiation [24–26], and epithelial-mesenchymal transition (EMT) [27], is one of the most mutated genes in HCC and a potent regulator of metastasis [27]. It is involved in regulating the mitochondrial apoptosis pathway [28], The results of our previous study showed that the overexpression of LASS2 over-elicited mitochondrial apoptosis in HepG2 cells [21], but the relation between LASS2 and p53 remains unclear.

Herein, the relationship between LASS2 and p53 is investigated, its prognostic value in liver cancer is assessed, and the underlying molecular mechanisms are investigated.

## Results

### Overexpression of LASS2 suppresses proliferation, invasion, and migration, and promotes apoptosis in liver cancer cell lines

**Fig. 1:**
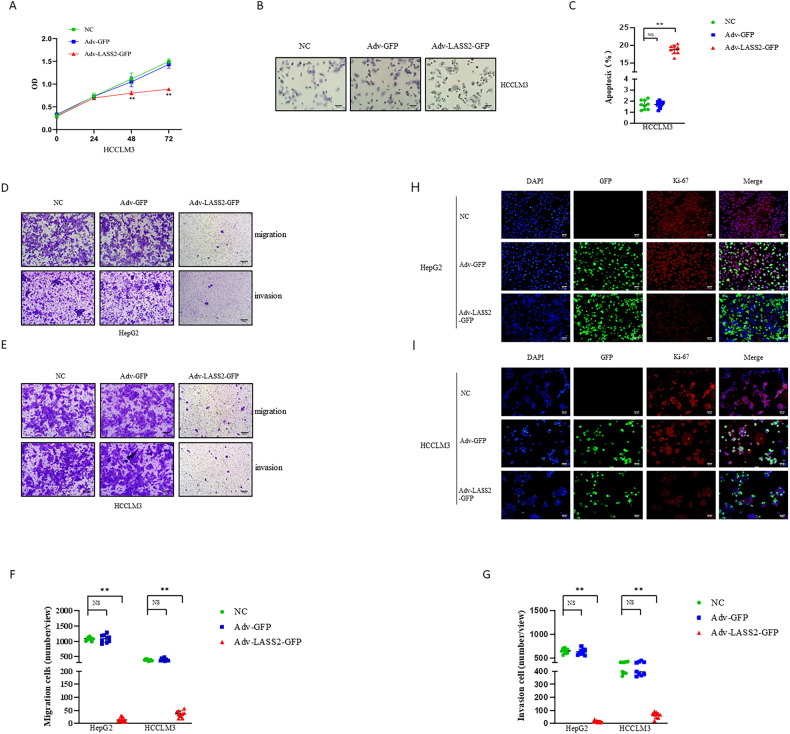
Overexpression of LASS2 suppresses liver cancer tumorigenicity in vitro. **A** CCK-8 assay for HCCLM3 cell line demonstrates that overexpression of LASS2 suppresses hepatoma cells proliferation. **B** Apoptotic DNA fragmentation in HCCLM3 cell line was detected using a dUTP TUNEL assay. Scale bar, 50 µm. Quantification results of TUNEL assay showed that overexpression of LASS2 promotes hepatoma cells apoptotic (**C**). Migration and invasion assay for HepG2 (**D**) and HCCLM3 (**E**) cell lines. The cell migration (**F**) and invasion (**G**) capability were significantly inhibited in LASS2-overexpressed hepatoma cells compared to negative control and Adv-GFP transfected cells. Immunofluorescence analysis for Ki67 in HepG2 (**H**) and HCCLM3 (**I**) cell lines. Scale bar, 50 µm (**P < 0.01, as indicated).

According to a previous report, the overexpression of LASS2 inhibits proliferation and induces apoptosis in HepG2 HB cells [21]. To investigate whether LASS2 also alters these biological characteristics of HCCLM3 HCC cells and HuCCT1 CCA cells, the CCK-8 and dUTP TUNEL assay were performed. The results consistently suggested that HCCLM3 and HuCCT1 cells with LASS2 overexpression displayed lower proliferation rates and could induce higher apoptosis cells compared with that in the negative or vector-control cells (all *P* < 0.05, Fig. 1A–C and Fig. S1A–C). The results from the Ki-67 immunofluorescence staining assay further indicated that LASS2 overexpression decreased the proliferation ability of HepG2, HCCLM3, and HuCCT1 cells (Fig. 1H, I and Fig. S1G).

The findings of the transwell migration and matrigel invasion assays revealed that LASS2 overexpression resulted in a lower migration and invasion capability compared with the negative control or vector-control cells in the liver cancer cell lines (all *P* < 0.01, Fig. 1D–G and Fig. S1D–F).

### Down-regulated expression of LASS2 predicted a poor prognosis of liver cancer in the test cohort

**Fig. 2:**
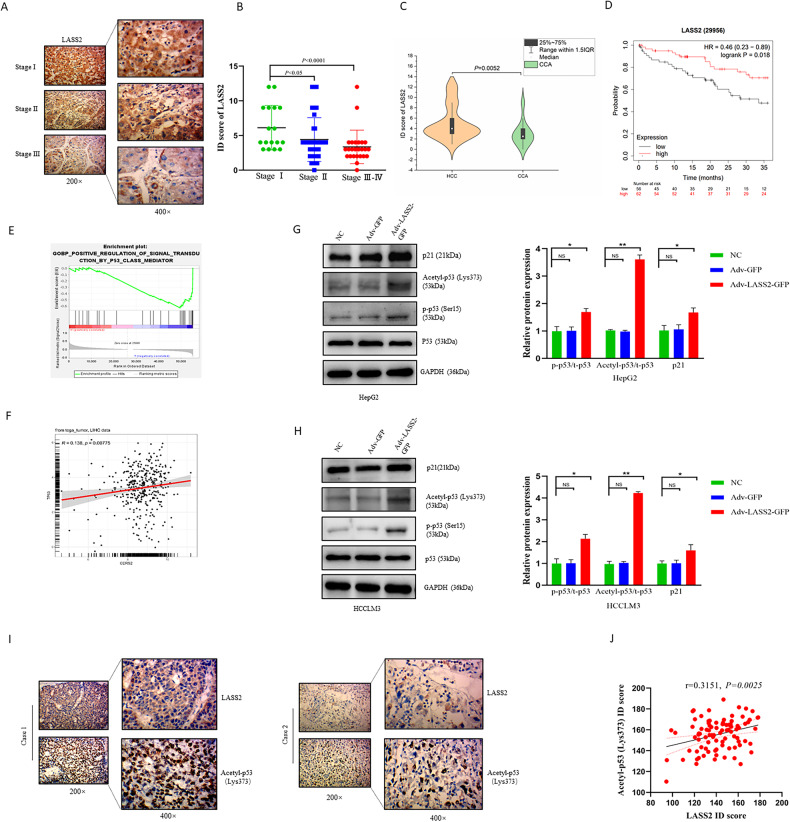
Low expression of LASS2 is correlated with liver cancer progression and p53 signaling pathway activation. **A** Representative IHC staining of LASS2 in liver cancer (HCC, *n* = 60; CCA, *n* = 30) different pathological stage (stage I (*n* = 17), stage II (*n* = 48), stage III–IV (*n* = 25)) and (**B**) scoring of LASS2 protein expression. **C** Violin plot analysis of LASS2 protein expression in HCC and CCA tumor tissue. **D** KM plotter of overall survival (OS) reveals the prognostic value of LASS2 mRNA expression in HCC patients. **E** Gene ontology (GO) analysis on biological process (BP) showed that LASS2-induced differentially expressed genes significantly were enriched in p53 signaling pathway. **F** A correlation analysis between LASS2 and TP53 mRNA expression based on TCGA-LIHC database. **G**, **H** Western blot analysis and quantification of total p53, p-p53 (Ser15), acetyl-p53 (Lys373) and p21 protein levels in HepG2 (**G**) and HCCLM3 (**H**) cell lines. **I** Representative IHC staining of LASS2 and acetyl-p53 (Lys373) in tumor tissue with HCC (*n* = 60) and CCA (*n* = 30) patients. **J** Statistic analysis of IHC staining indicates that LASS2 expression is positively correlated acetyl-p53 (Lys373) (*P < 0.05, **P < 0.01, as indicated).

To investigate the role of LASS2 in liver cancer progression, 90 liver cancer specimens (the test cohort consists of 60 HCC patients and 30 human CCA patients) were collected and stained using immunohistochemistry (IHC) to assess LASS2 protein expression. The results of IHC staining analysis revealed that LASS2 protein is relatively abundant in the early-stage disease (TNM stages I or II) and scarce in later-stage liver cancer (TNM stages III and IV) (Fig. 2A). Statistical analyses, based on the semi-quantification of the IHC staining (ID score), indicated that LASS2 expression level was negatively correlated to the TNM stage (*P* = 0.013, Table 1) (TNM stages II vs. I: *P* = 0.011; TNM stages III–IV vs. I: *P* < 0.001, Fig. 2B) and tumor size (*P* = 0.029, Table 1), and lower LASS2 expression in CCA tumor samples compared to HCC tissue by violin plot analysis (*P* = 0.0052, Fig. 2C). As shown in Table 1, sex, age and the concentration of AFP in the patient serum were not significantly correlated to LASS2 expression levels. Using the KM plotter database including 118 tumor samples in HCC patients (*P* = 0.018, Fig. 2D), Kaplan–Meier survival curves revealed that liver cancer patients with lower LASS2 expression had significantly reduced overall survival.

**Table 1:** Relationships of LASS2 expression level with the clinicopathological features in liver cancer patients (*n* = 90).

Characteristics	Lass2 ID Score		*P*
	0-4	6-12	
Sex			0.086
Male	49	19	
Female	20	2	
Age (years)			0.798
≤50	27	7	
>50	42	14	
Tumor size			0.029^*^
≤30 mm	4	5	
>30 mm	65	16	
TNM Stage			0.013*
I	9	8	
II	37	11	
III–IV	23	2	
AFP			0.803
≤20 ng/ml	43	11	
>20 ng/ml	26	10	
Acetyl-p53(Lys-373)			0.033*
Low expression	51	10	
High expression	18	11	

*P < 0.05.

### LASS2 induces the phosphorylation and acetylation of p53 and activates the p53 signaling pathway

As shown by the GeneSet enrichment analysis (GSEA), the LASS2-induced differentially expressed genes were enriched in the p53 signaling pathway (Gene Ontology Biological Process, GOBP analysis) (*P* = 0.006, FDR *q* = 0.591, Fig. 2E). An analysis of TCGA-LIHC (Liver hepatocellular carcinoma) dataset from GTBAdb online tools indicated the significant and positive correlation of LASS2 (CERS2) expression levels with TP53 mRNA expression (*r* = 0.138, *P* = 0.008, Fig. 2F). Therefore, it was further investigated whether LASS2 affects the signaling pathway of p53. The western blotting results (Fig. 2G) revealed that LASS2 overexpression did not alter total p53 levels. Interestingly, the enhanced phosphorylation of p53 (at ser15) and acetylation of lys373 (acetyl lys373), and increased p53 directly targeting genes such as p21 were observed in LASS2-overexpressing TP53 wild-type cell line HepG2 cells (Fig. 2G). Similarly, in LASS2-overexpressing TP53 mutant cell lines (HCCLM3 and HuCCT1), p53 phosphorylation and acetylation still occurred, and the p21 protein level was also upregulated (Fig. 2H and Fig. S2A).

The correlation between LASS2 and acetylation of p53 at lys373 (acetyl lys373) was confirmed using 60 HCC and 30 CCA. Similar results were observed in clinical liver cancer sections by IHC, and the LASS2 protein levels were also found to be positively correlated with acetylation of p53 (lys373) expression (*r* = 0.3151, *P* = 0.003, Fig. 2I, J). Collectively, these results indicate that the overexpression of LASS2 activates the p53 signaling pathway.

### LASS2 promotes p53-mediated mitochondrial apoptosis in liver cancer cells

**Fig. 3:**
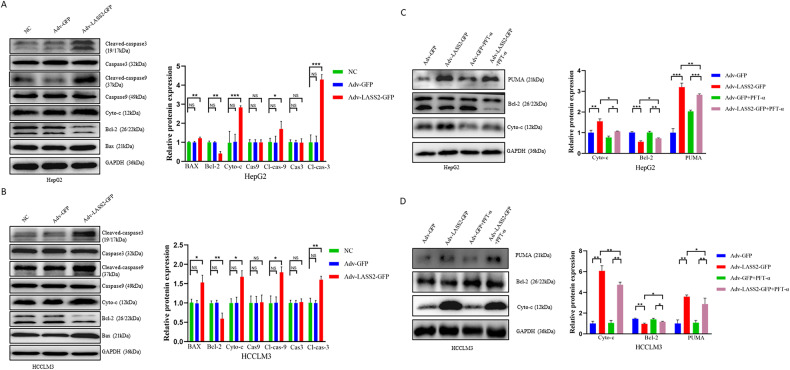
LASS2 overexpression induces p53-mediated mitochondrial apoptosis in liver cancer cells. **A**, **B** Representative immunoblots and quantitative analysis of Bax, Bcl-2, Cyto-c, caspase-3, 9, and cleaved-caspase-3, 9 in HepG2 (**A**) and HCCLM3 (**B**) cell lines. **C**, **D** Western blot (left) and quantification (right) of PUMA (a critical target for p53-mediated apoptosis), Bcl-2, Cyto-c expression in HepG2 (**C**) and HCCLM3 (**D**) cells either untreated or pretreated with p53 inhibitor PFT-α (20 μM) and following transfected with Adv-GFP or Adv-LASS2-GFP (*P < 0.05, **P < 0.01, ***P < 0.001, as indicated).

To determine the role of p53 in mitochondrial apoptosis, the proteins involved in p53-mediated apoptosis were further assessed by western blot. The western blotting results also confirmed the close association of LASS2 with mitochondrial apoptosis in HepG2, HCCLM3, and HuCCT1 cell lines. The overexpression of LASS2 reduced Bcl-2 expression while upregulating the protein levels of Bax, Cyto-c, Cleaved-caspase-3, and Cleaved-caspase-9 (all *P* < 0.05, Fig. 3A, B and Fig. S2B). As PUMA is a downstream target gene of p53 and promotes apoptosis through p53-dependent and p53-independent pathways, and given the crucial role for PUMA and Cyto-c, Bcl-2 mediated mitochondrial apoptotic pathway, we examined whether these pathways may also promote apoptosis in response to p53-inhibition by pifithrin-α (PFT-α). As shown in Fig. 3C, D and Fig. S2C, after PFT-α treatment, overexpression of LASS2 attenuated PUMA and Cyto-c, Bcl-2 mediated mitochondrial apoptosis, downstream target of p53.

### LASS2 impedes EMT and ECM in liver cancer cell lines through the p53 signaling pathway

**Fig. 4:**
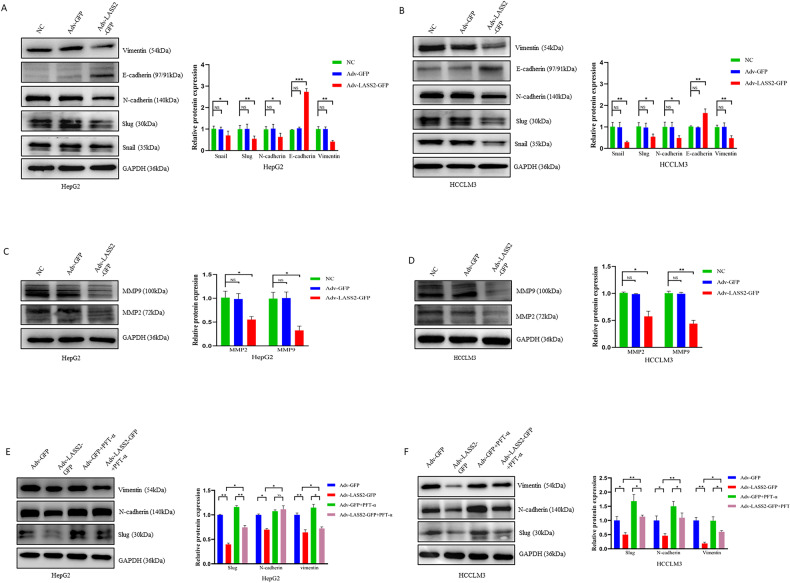
LASS2 inhibits EMT and ECM in liver cancer cell lines through the p53 signaling pathway. **A**, **B** Representative western blot images (left) and quantification (right) of EMT markers (snail, slug, N-cadherin, E-cadherin, and vimentin) in (**A**) HepG2 and (**B**) HCCLM3 cell lines. **C**, **D** The expression of ECM markers (MMP2 and MMP9) were detected by western blotting and quantified by western blot analysis in HepG2 (**C**) and HCCLM3 (**D**) cells. **E**, **F** Representative western blot of cell-lysates from (**E**) HepG2 with WT p53 or (**F**) HCCLM3 with p53 mutant status, indicated that LASS2 overexpression inhibits p53 downstream EMT targets (slug, N-cadherin, and vimentin), at least in part, through the p53 signaling pathway (*P < 0.05, **P < 0.01, ***P < 0.001, as indicated).

To further study the mechanism by which LASS2 regulates cell migration and invasion, the protein levels of EMT markers in LASS2 overexpressed cell lines and control cell lines were determined. The results showed that LASS2 overexpression increased the protein expression of epithelial cell markers (E-cadherin), while decreasing the expression of mesenchymal cell markers (N-cadherin, vimentin) and EMT transcriptional drivers (Snail, Slug) in HepG2, HCCLM3, and HuCCT1 cells (all *P* < 0.05, Fig. 4A, B and Fig. S2D). Two matrix metalloproteinases (MMPs) MMP2 and MMP9 are the endopeptidases involved in ECM remodeling, EMT, and tumor cell invasion [29–31]. Consistent with an EMT phenotype, HepG2, HCCLM3, and HuCCT1 cells with LASS2 overexpression showed the diminished degradative capabilities of ECM, as indicated by their reduced MMP2 and MMP9 expressions (all *P* < 0.05, Fig. 4C, D and Fig. S2E). Importantly, the effect of LASS2 overexpression on the expression of p53 downstream EMT targets (Slug, N-cadherin, and vimentin) in *WT* p53 and mutant p53 hepatoma cell lines (*P* < 0.05, Fig. 4E, F and Fig. S2F) was also found to be attenuated by the p53 inhibitor PFT-α.

### LASS2 directly interacts with MDMX/MDM2

**Fig. 5:**
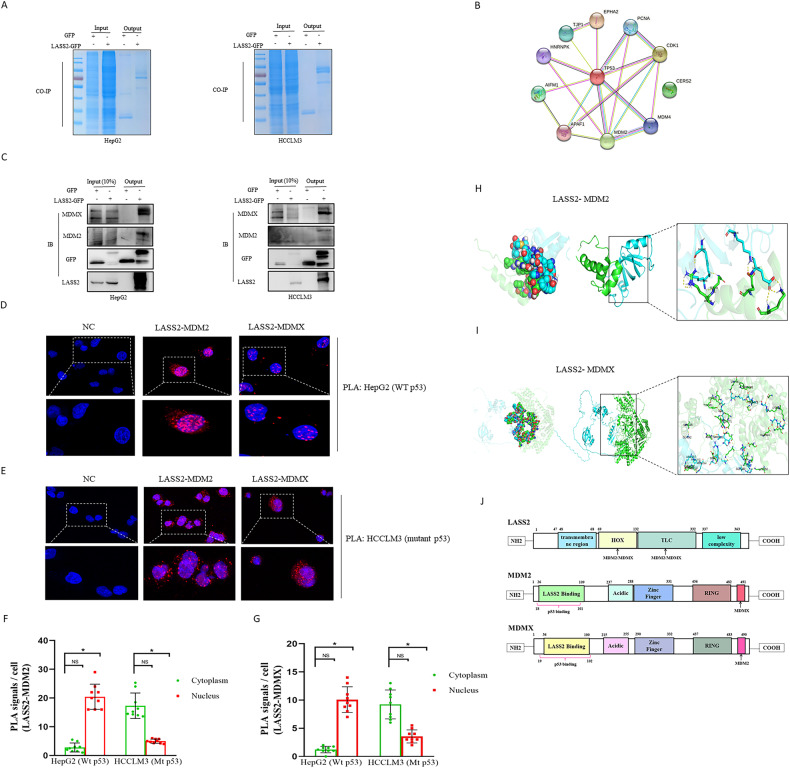
LASS2 directly interacts with MDMX/MDM2. **A** GFP-precipitated bound proteins were visualized by staining Coomassie after SDS-PAGE. **B** Visualization using STRING of the net-work of interactions between LASS2 binding proteins related the p53 signaling pathway in Hepa1-6 hepatoma cells. **C** Western blot analysis following Co-IP experiments using HepG2 and HCCLM3 cell lines validated the interaction of LASS2 and MDM2 or MDMX. **D** Representative pictures of proximity ligation assay (PLA) in situ revealed endogenous interactions between LASS2 and MDM2 or LASS2 and MDMX in HepG2 (WT p53) and in (**E**) HCCLM3 (mutant p53) cell lines (red dots). **F** Quantification for number of PLA positive signals co-localized in the nucleus and cytoplasm per cell revealed that major nucleus interactions between LASS2 and MDM2 or (**G**) LASS2 and MDMX in HepG2 with wild-type p53 status and major cytoplasmic interactions in HCCLM3 with mutant p53 status cell lines. **H** Protein–protein docking between LASS2 and MDM2 or (**I**) MDMX was visualized by the web server ClusPro 2.0. The surface of LASS2 is colored in green, and MDM2 or MDMX is colored in blue. Yellow dashed line represents hydrogen bonding interactions. **J** Schematic diagram of the binding sites of LASS2 to MDM2 or MDMX (*P < 0.05, as indicated).

The capability of LASS2 to significantly influence liver cancer cell line phenotype has been demonstrated in the current study. However, the underlying molecular mechanism remains unknown to date. Further research is needed to clarify the regulatory mechanisms of LASS2 in the inhibition of liver cancer progression, the possible LASS2 interaction proteins were identified by co-immunoprecipitation (co-IP)-coupled Liquid Chromatography-Mass Spectrometry (LC/MS) in Hepa1-6 hepatoma cells. The higher number of differential bands specific to LASS2-GFP in the coomassie blue stained SDS-PAGE gel was observed as compared to the GFP control, as shown in Fig. 5A and Fig. S3A. Protein interaction analysis (PIP) demonstrated that the p53 signaling pathway-related proteins might interact with LASS2, including APAF1, MDM2, MDMX, CDK1, PCNA, EPHA2, TJP1, HNRNPK, and AIFM1 (Fig. 5B). Notably, both MDM2 and MDMX are the critical negative regulators of p53 function [32, 33]. To determine the direct interaction between LASS2 and MDM2 or MDMX, their interactions were studied by co-IP using GFP Nanoselector beads for immunoprecipitation in TP53 wild-type HepG2 and TP53 mutant HCCLM3 and HuCCT1 cell lines, followed by western blot with anti-GFP, anti-LASS2, anti-MDM2, and anti-MDMX. Co-IP western blot results confirmed the presence of interactions between LASS2 and MDM2 or MDMX (Fig. 5C and Fig. S3B). Proximity Ligation Assays (PLAs) further confirmed these findings (Fig. 5D–G). Significant PLA signals were detected in HepG2 (WT p53) and HCCLM3 (mutant p53) cells, and the PLA interaction signals of LASS2 with MDM2 or MDMX were primarily localized in the nucleus of HepG2 (WT p53) but in the cytoplasm of HCCLM3 (mutant p53) cells.

Therefore, protein–protein docking studies were performed to gain insights into the nature of molecular interactions between LASS2 with MDM2 or MDMX. The docking results were obtained by selecting the lowest energy model from the ten models with low binding energies according to their Weighted Scores. The contact lists between LASS2 with MDM2 or MDMX are shown in Table S1–3. Next, the protein interaction binding modes and sites between these proteins were predicted using PyMol software. The results of docking studies in the LASS2-MDM2 or MDMX binding models are shown in Fig. 5H, I and Tables S2, 3. Domains are considered to be the structural and functional units of a protein, LASS2 protein has distinct Homeobox-like domains (amino acids 71–128), Tram-Lag-CLN8 (TLC) domains (amino acids 131–332), and Lag1p motifs. By using ZDOCK 3.0.2 to predict functional protein domains, LASS2 was found to probably interact with MDM2 or MDMX through its Homeobox-like domains and TLC domain (Fig. 5J). Interestingly, the binding sites of LASS2 and MDM2 or MDMX overlap with the previously reported [34] binding sites of p53 and MDM2 or MDMX (Fig. 5J).

### LASS2 inhibits MDM2/MDMX expression and enhances p53 stability in liver cancer cells

**Fig. 6:**
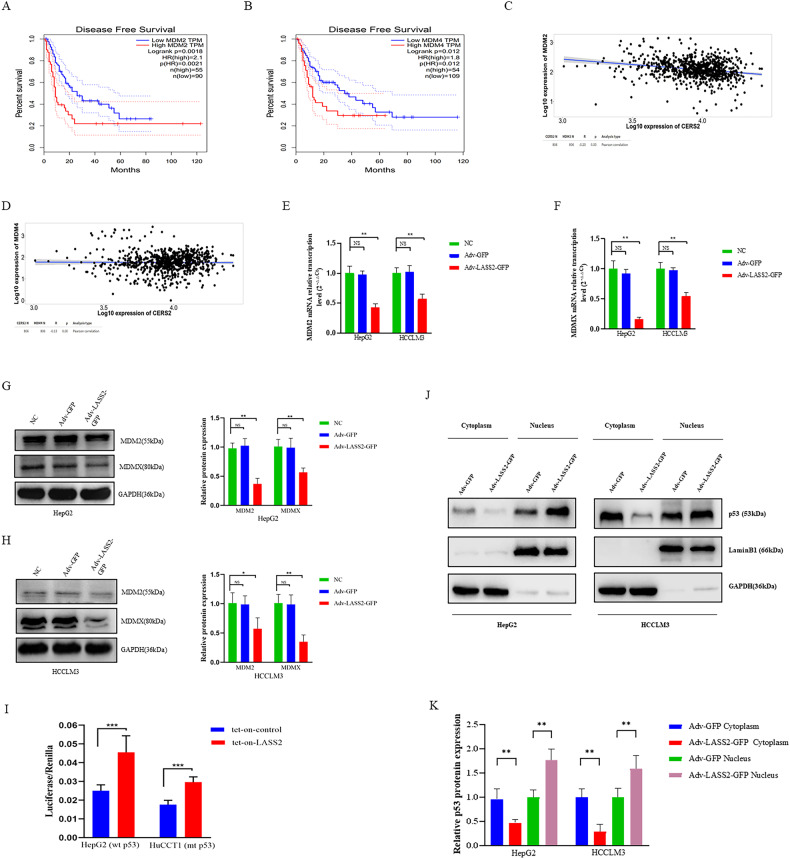
LASS2 inhibits MDM2/MDMX expression and enhances p53 stability in hepatoma cells. **A**, **B** Disease Free Survival (DFS) analysis of (**A**) MDM2 and (**B**) MDMX expression in patients with liver cancer using the Kaplan–Meier Plotter database. **C**, **D** A analysis of the relationship between LASS2 and (**C**) MDM2 or (**D**) MDMX demonstrated that LASS2 transcript levels were negatively correlated with MDM2 or MDMX in the TNMplot database (https://www.tnmplot.com/). **E**, **F** LASS2 overexpression inhibits the mRNA levels of (**E**) MDM2 or (**F**) MDMX in HepG2 and HCCLM3 cells. **G**, **H** Representative western blotting images (left) and quantification (right) of MDM2 and MDMX in (**G**) HepG2 and (**H**) HCCLM3 cell lines. **I** Dual luciferase assays show that LASS2 overexpression increase the transcriptional activity of p53. **J**, **K** Representative western blotting images (**J**) and quantification (**K**) of p53 protein in the nucleus and cytoplasm of HepG2 with *WT* p53 or HCCLM3 with p53 mutant cells (*P < 0.05, **P < 0.01, ***P < 0.001, as indicated).

As shown in Fig. 6A, B, using the Kaplan–Meier Plotter database (MDM2 DFS: Hazard ratio [HR] = 2.1, *P* = 0.0018; MDMX DFS: HR = 1.8, *P* = 0.012), multivariable analysis for Disease Free Survival (DFS) determined that high MDM2 or MDMX expression is associated with shorter DFS in liver cancer patients. To further elucidate the regulatory relationship between LASS2 and MDM2 or MDMX in the process of liver cancer propagation, an online TNMplot database was used to conduct a computational analysis of these relationships in LIHC. The results demonstrated that LASS2/CERS2 transcript levels were negatively correlated with MDM2 or MDMX expression (MDM2: *r* = −0.20, *P* < 0.01; MDMX: *r* = −0.13, *P* < 0.01, Fig. 6C, D). In addition, LASS2 overexpression was found to inhibit the mRNA and protein expression levels of MDM2 or MDMX in HepG2, HCCLM3, and HuCCT1 cells (all *P* < 0.01, Fig. 6E–H and Fig. S3C–E), respectively. As the dual inhibition of MDMX and MDM2 is required for the full release of dormant p53 [33], dual inhibition is a potential pathway for p53 activation [35]. Dual luciferase reporter assays were performed to investigate whether LASS2 regulates the p53 signaling pathway. A remarkable increase in the transcriptional activity of p53 was observed by LASS2 overexpression (Fig. 6I). Furthermore, the p53 protein levels in the nucleus and cytoplasm were studied. As shown in Fig. 6J, K and Fig. S3F, the overexpression of LASS2 stimulated the nuclear translocation of p53 in TP53 wild-type cell lines and TP53 mutant cell lines. Collectively, these findings further indicated that LASS2 activates the p53 signaling pathways and enhances p53 stability in HepG2, HCCLM3, and HuCCT1 cells.

## Discussion

HCC, CCA, and HB collectively represent the major forms of liver malignancies with high recurrence and metastasis rates and are malignancies with poor prognosis [36–39]. Abnormal gene expression is significantly associated with the occurrence and inadequate prognosis of these three liver cancer subtypes [40]. Although low LASS2 expression in tumor tissues of HCC patients is associated with prognosis [22, 23], a limited understanding of its expression in other liver cancer subtypes and the underlying molecular mechanisms is available.

In the current work, functional studies revealed that LASS2 inhibited the proliferation, apoptosis, invasion, and migration of different subtypes of liver cancer cell lines. Next, LASS2 expression was found to be down-regulated in patients with HCC and CCA, which correlated with their poor prognosis. Bioinformatics predictions indicated that high LASS2 expression was associated with the p53 signaling pathway, which was experimentally verified.

The p53 protein is a sequence-specific DNA-binding transcription factor encoded by the TP53 gene and is known as the “guardian of the genome” [41]. p53 activity is usually regulated at the protein level by post-translational modifications (PTM) [42]. Under stress-free conditions, p53 protein levels are usually maintained low by continuous degradation. In stressed cells, the p53 protein is subject to various post-translational modifications that affect the expression of p53 target genes, including phosphorylation and acetylation [42].

**Fig. 7:**
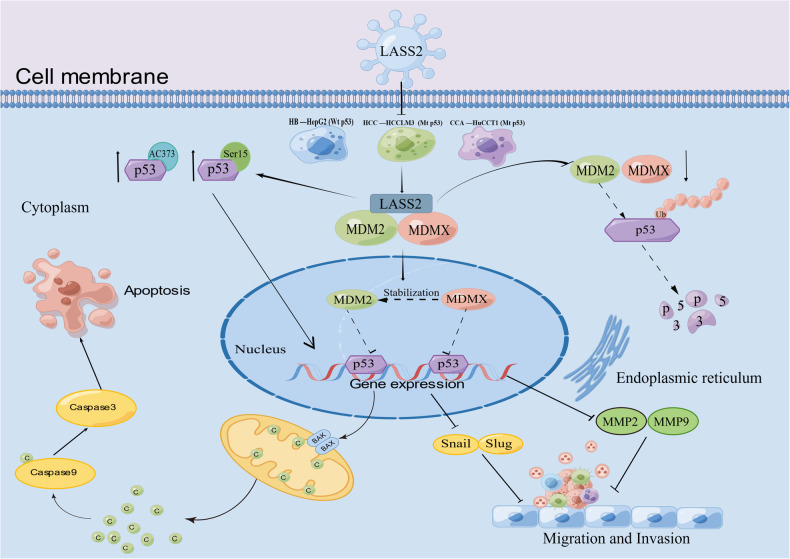
Schematic illustration of the molecular mechanism by which LASS2 inhibits the liver cancer progression. LASS2 exerts its tumor-suppressive effects in a p53-dependent manner, in which LASS2 interacts with MDM2/MDMX and causes dual inhibition to disrupt p53 degradation by MDM2/MDMX.

The data from the current study clearly show that LASS2 overexpression facilitates the phosphorylation of p53 at ser15 and acetylation of p53 at lys373 and increases the protein expression of the key downstream target gene p21 in TP53 wild-type HepG2 and mutant HCCLM3 and HuCCT1 cell lines. Additionally, LASS2 overexpression promoted the transcriptional activity of p53, as revealed by the dual luciferase reporter system. Therefore, LASS2 is suggested to regulate p53 activation through a two-pronged approach. Subsequent Cox proportional risk regression model analysis obtained from TCGA-LIHC further demonstrated the positive correlation of LASS2 expression with TP53 mRNA expression levels. Owing to the unstable expression and rapid degradation of wild-type p53, it cannot be detected by the IHC method [43]. In addition, acetylation is critical for p53 because it enhances p53 protein stability [44], binds to other proteins, and is required for its checkpoint response to stress [45]. Therefore, the correlation between LASS2 and acetylated p53 was analyzed in clinical samples and the expression of LASS2 was positively associated with the level of acetylated p53 (lys373) protein expression. Collectively, the results of the present study support the association between LASS2 and activation of the p53 signaling pathway (Fig. 7). Further, LASS2 is found to exhibit a direct interaction with MDM2 and MDMX, and LASS2 overexpression is found to suppress the expressions of MDM2 and MDMX. MDM2 is a ubiquitin ligase that targets and degrades p53 through the ubiquitin-proteasome system [33]. MDMX, also known as MDM4, is a structural homolog of MDM2, but it lacks intrinsic E3 ubiquitin ligase binding activity. Moreover, it primarily inhibits p53 transcriptional activity but does not affect its protein stability [46, 47]. Several groups have reported that MDM2 and MDMX play indispensable and non-overlapping roles in inhibiting the normal function of p53, and dual inhibition by MDM2 and MDMX is essential for the full release of dormant p53 and is a potential pathway for p53 activation [33, 48, 49]. Given that the possible binding domains of LASS2 and MDM2 or MDMX (protein–protein docking in this study) share the same region as the binding domains of p53 and MDM2 or MDMX, it can be speculated that LASS2 may inhibit p53 degradation by competitively inhibiting the binding of MDM2 or MDMX to p53.

The activated tumor suppressor p53 can regulate the expression of dozens of target genes and different physiological processes [50]. The phosphorylation of p53 is a key modification that directs its regulation of apoptotic cell death, where phosphorylation at the ser15 site stabilizes p53 by protecting it from MDM2 [42]. The acetylation of the C-terminus of p53 (including lys53, lys370, lys372, lys373, lys381, and lys382) facilitates its binding to target gene sites to activate downstream pathways, and acetylation of these sites prevents the degradation of p53 and induces its accumulation [42, 45, 51, 52]. This study demonstrates that LASS2 overexpression contributes to the stabilization and accumulation of p53 protein in the nucleus of wild-type and mutant hepatoma cell lines, enhances the role of p53 in the regulation of downstream target gene activation or inhibition, and is involved in causing apoptosis (Bcl-2, Bax, Cyto-c, Cleaved caspase-3 and 9), EMT (Snail, Slug, N-cadherin, E-cadherin and vimentin), and ECM (MMP2 and MMP9) genes. Notably, HCCLM3 and HuCCT1 have mutant p53 status, but both express p53 (compared with HepG2, primarily expressed in the cytoplasm), and it was speculated whether LASS2 depends on p53 for its oncogenic effects when LASS2 overexpression exhibits the same biological role in these three cell lines with different p53 status. Unexpectedly, the p53 inhibitor pifithrin-α attenuated the effect of LASS2 overexpression on p53 downstream mitochondrial apoptosis target gene (PUMA), Bcl-2, Cyto-c, and also on EMT target genes (Slug), N-cadherin, and vimentin. The finding of this study suggests that LASS2 exerts a tumor suppressive function, at least in part, through the p53 signaling pathway in both wild-type and these two p53 mutant types of cell lines.

In summary, the results of the current study demonstrate for the first time that LASS2 exerts a tumor-suppressive effects by interacting with MDM2 and MDMX and promoting p53 ser15 phosphorylation and lys373 acetylation in the different subtypes of hepatoma cells that harbor wild-type and mutant p53, thereby stabilizing p53. As we all know, p53 activation is an attractive strategy for anticancer therapy. These findings highlight the importance of the LASS2-MDM2/MDMX-p53 signaling axis in the regulation of liver cancer and may elucidate the mechanisms of mutant p53 reactivation, providing possible new targets for clinical treatment.

## Materials and methods

### Cell culture and transfections

Human HCC cell line HCCLM3 (mutant p53) and human Cholangiocarcinoma (CCA) cell line HuCCT1 (mutant p53, R175H) were obtained from Feng Hui Biotechnology company (Changsha, Hunan, China). Human HB cell line HepG2 (wild-type p53) and mouse hepatoma cell line Hepa1-6 were kindly provided by Stem Cell Bank, Chinese Academy of Sciences (Shanghai, China). All cell lines were routinely cultured in DMEM medium (Gibco) or RPMI-1640 (HuCCT1) (Gibco) containing 10% (v/v) fetal bovine serum (FBS) and 1% penicillin/streptomycin at 37 °C with 5% CO_2_.

The recombinant human adenovirus vector overexpressing LASS2 (Adv-*h*LASS2-GFP), the recombinant mouse adenovirus-expressing LASS2 vector (Adv-*m*LASS2-GFP), and the control adenovirus vector (Adv-GFP) were constructed as described in a previously reported study [21].

HCCLM3, HuCCT1, and HepG2 cells were transfected with Adv-GFP or Adv-*h*LASS2-GFP for 48 h, or the cells were treated with PFT-α (20 μM, MCE) for 24 h and then transfected for 48 h, respectively. Next, the cells were separately harvested according to the requirement of each experiment, and all assays were independently performed in triplicates.

### Cell viability assay

A CCK-8 assay (Solarbio, Beijing, China) was performed to determine cellular proliferation. A total of 6000 cells were seeded into 96-well plates (Corning). The plate was incubated overnight with 5% CO_2_ at 37 °C. At 24, 48, and 72 h post-transfections with Adv-GFP or Adv-*h*LASS2-GFP, CCK-8 reagent was added into each well. Using a microplate reader (Thermo Multiskan, USA), the optical density of the solution measurement at 450 nm was performed after incubation for 2 h.

### Cell migration and invasion assay

Transwell chambers (8 μm pore size, 6.5 mm diameter, Corning Costar) were used to study cell migration and invasion abilities. A total of 5 × 10^4^ cells/mL were added to the upper chamber with serum-free medium. Then, a DMEM medium containing 20% FBS was placed in every well of the lower chamber. After incubating for 48 h, the cells were fixed with 4% paraformaldehyde and stained with 0.1% crystal violet ammonium oxalate solution (Solarbio, China), and the remaining cells on the upper surface were wiped with cotton balls. Finally, under a microscope (Nikon, Japan), the stained cells that had migrated through the transwell polycarbonate membrane were observed. Diluted Matrigel (at a ratio: of 1:8) was used in the invasion assay.

### Terminal deoxynucleotidyl transferase dUTP nick end labeling (TUNEL) assay

TUNEL, a terminal deoxynucleotidyl transferase-dUTP nick end labeling assay (KeyGen Biotech, China), was used to detect cell apoptosis. Briefly, cells are fixed, permeabilized, labeled, ligated, and stained, and then the number of brown-stained cell nuclei were counted under a microscope (Olympus, Japan).

### Immunofluorescence (IF) staining

After the cells were fixed and permeabilized, the cells were blocked with 10% goat serum and incubated overnight at 4 °C with a primary antibody against Ki-67 (Proteintech, 273099-1-AP, 1:1000). The cells were incubated with CoraLite594-conjugated goat anti-rabbit IgG (Proteintech, SA0013-4, 1:100). For the final step, the cells were incubated with Hoechst 33342 (CST, 4082S, 1:1000) for 15 min. Dark conditions were maintained and the cells were observed under a fluorescent microscope (Olympus, Japan).

### Human clinical samples

Ninety formalin-fixed, paraffin-embedded tissues were obtained from primary liver cancer patients who underwent surgery and were untreated with radiotherapy/chemotherapy in the Affiliated Hospital of ZunYi Medical University (Guizhou, China). All samples were acquired after obtaining patient consent and approval from the Ethics Committee of the Affiliated Hospital of ZunYi Medical University (Ethics approval number: KLL-2019-020). As shown in Table 1, the detailed clinical data of patients are provided.

### Immunohistochemistry (IHC)

Sixty human HCC and 30 human CCA tissue paraffin sections (4-µm thick) were deparaffinized and IHC was carried out as described previously [53]. The complete list of primary and secondary antibodies and related information is provided in Table S1. The score for staining was independently assessed by two experienced and blinded observers based on the staining intensity and percentage of positive cells. The staining intensity was scored as follows: cells with no positive staining were defined as 0 points; 1 point for light yellow; 2 points for yellow or light brown; cells with dark brown staining were defined as 3 points. The percentage of positive cells was scored as follows: cells with less than 5% staining were scored as 0 points; cells with 5–25% staining were scored as 1 point; the score of 26–50% stained cells was 2 points; 51–75% positively staining cells were scored as 3 points; and that with 76–100% were scored as 4 points. These two types of points were multiplied to generate overall scores (ID scores). The sum of the average integrated optical density (IOD) of each sample was calculated using Image J software.

### Real-time PCR and western blotting

Following the total RNA extraction with RNAiso Plus reagent (Takara, Japan), and the total protein fractions of HCCLM3, HuCCT1, and HepG2 cells were extracted with a protein extraction kit (KeyGen Biotech, China). A nuclear and cytoplasmic protein extraction kit (APPLYGEN, China) was used to extract nuclear and cytoplasmic proteins of the liver cancer cell lines for detecting p53 protein. Protein concentration was measured using the BCA reagent kit (Epizyme Biotech) according to manufacturer’s instructions. All these procedures were performed as previously described [54]. The primers and antibodies are listed in Tables S4 and S5.

### Co-immunoprecipitation (Co-IP) LC-MS/immunoblotting (IB)

Hepa1-6 hepatoma cells were transfected with Adv-GFP or Adv-*m*LASS2-GFP for 48 h, and then the cells were lysed with ice-cold IP lysis/wash buffer (Thermo Scientific Pierce, USA) after washing twice with PBS. As instructed by the manufacturer (GFP Nanoselector Agarose one-step immunoprecipitations kit, NBbiolab, China), co-IP experiments were performed as described previously [55]. Briefly, to capture GFP-fusion proteins, lysates were incubated for 1 h at 4 °C with 25 μL equilibrated GFP Nanoselector beads. Thereafter, the beads were washed with wash buffer, resuspended in 2 × SDS-sample buffer, and boiled for 10 min at 95 °C to dissociate the immuno-complexes from the beads. Using SDS-PAGE, proteins were separated, and stained with Coomassie Blue (Epizyme, China), and then the stained SDS gel portions were excised, destained, dehydrated, dried, and digested with trypsin. Subsequently, freeze-dried tryptic peptides were subjected to LC-MS analysis. An online tool called STRING (*ver*. 11.5) (https://cn.string-db.org/) was used to identify the potential interaction networks and functional enrichments of these interactions of proteins (gene ontology (GO) annotation, KEGG pathway).

The Co-IP samples of HCCLM3, HuCCT1, and HepG2 cells transiently transfected with Adv-GFP and Adv-*h*LASS2-GFP were analyzed by western blotting to determine the interaction between LASS2 and MDMX or MDM2.

### Proximity ligation assay

A PLA kit (Duolink, Sigma Aldrich) was used to detect LASS2 and MDM2 or MDMX interactions. In brief, after the cells were fixed and permeabilized, they were permeabilized with 0.1% TRITON X-100 and incubated with Duolink^®^ Closure Solution. Anti-LASS2 (1:500, sc-390745, Santa cruz, American) and anti-MDM2 (1:200, ER1902-14, HuaBio, China) or MDMX (1:500, 17914-1-AP, Proteintech, China) were mixed and diluted in Duolink^®^ antibody diluent and incubated overnight at 4 °C. The PLUS and MINUS PLA probes were mixed and diluted in Duolink^®^ antibody diluent (dilution ratio 1:5) and incubated for 1 h at 37 °C in a preheated humidity chamber. After ligation, amplification, and washing, the slices were sealed with Duolink^®^ in-situ sealer containing DAPI, waited for 15 min to acquire images under a confocal microscope (Leica SP8, Germany), and analyzed with the confocal software.

### Protein–protein docking studies

The amino acid sequences of LASS2 and interacting proteins MDMX or MDM2 were downloaded from the RCSB PDB database (https://www.rcsb.org/). To create the 3D models of these proteins, homology modeling was performed using SWISS-MODEL (https://swissmodel.expasy.org/). Subsequently, a set of the highest predictive-performing models and their modeling conditions were selected. Protein–protein docking was performed using the web server ClusPro 2.0 (https://cluspro.org) for these two protein structures to predict their binding interaction. Based on the lowest Weighted Score, the docking structure was visualized with the PyMol software (version 2.5, available at http://www.pymol.org).

### Bioinformatics

The transcriptome data and corresponding clinical information of HCC patients were obtained from The Cancer Genome Atlas (TCGA) (http://cancergenome.nih.gov/), including 374 tumor and 50 normal liver samples. The “limma” R package was used to analyze the differential expression of specific genes between cancer and normal liver tissues, and the relationship between clinical features and gene expression was also evaluated by R software. GSEA was performed to investigate the functions of LASS2 based on the TCGA database with the “Clusterprofile” package. *P* < 0.05 was considered to be statistically significant enriched function annotations in Gene Ontology Biological Process (GOBP) pathways. Gene correlation analysis was performed through the online tools GTBAdb analysis (https://www.gtbadb.com/) or TNM plot analysis (https://www.tnmplot.com/). According to the Kaplan–Meier Plotter database (http://kmplot.com/analysis/), survival curves were plotted and analyzed using log-rank tests. A published study consisting of RNA-seq data and clinical information of 118 HCC patients available at the KM plotter was used to assess overall survival against the LASS2 mRNA data.

### Dual-luciferase reporter assay

The HepG2 (wt p53) and HuCCT1(mutant p53) cells stably expressing doxycycline-inducible constructs of LASS2 were pretreated with doxycycline for 48 h before seeding, and then were plated in 24-well plates at 1.5 × 10^5^ cells per well before the transfection of luciferase reporter plasmids. PG13-luc luciferase reporter and pRL-CMV plasmid (Renilla luciferase, Promega) were cotransfected into hepatoma cells as mentioned above. As directed by the manufacturer, the activity of luciferase was determined with a dual luciferase reporter assay system (Promega).

### Statistical analysis

All data analyses were performed using GraphPad Prism 8.0. Differences in categorical variables were assessed by χ^2^ tests, and differences in continuous variables were measured by applying one-way ANOVA. Based on Pearson’s correlation analysis, the relationship between the variables was determined. *P*-values < 0.05 were considered to be statistically significant (*P < 0.05; **P < 0.01; ***P < 0.001); *P* > 0.05 was considered non-significant (NS).

All bioinformatics analyses of data downloaded from the TCGA database were performed using statistical software R (version 4.0.3).

### Supplementary information

Figure legeneds for supplementary

Figure. S1

Figure. S2

Figure. S3

Supplemental table 1-5

## Data Availability

All data used or analyzed during this study are included in this article and available from the corresponding author upon reasonable request.
