# Supplementary material for: LASS2 enhances p53 protein stability and nuclear import to suppress liver cancer progression through interaction with MDM2/MDMX
Source: Cell Death Discov. 2023 Nov 14;9:414. doi: 10.1038/s41420-023-01709-2 (PMC10646090; doi:10.1038/s41420-023-01709-2)
Supplement: Supplementary file 1 — Figure legeneds for supplementary [file 41420_2023_1709_MOESM1_ESM.docx]

**Figure S1**

**A** CCK-8 assay for HuCCT1 cells. **B** A dUTP TUNEL assay for detecting apoptotic DNA fragmentation in HuCCT1 cells. Scale bar, 50 µm. **C** Quantitative analysis of TUNEL -positive cells in different experimental groups of HuCCT1 cell line. **D** Transwell migration and invasion assay for HuCCT1 cells. **E, F** LASS2 overexpression significantly impaired the migration **(E)** and invasion **(F)** ability of HuCCT1 cell line. **G** Immunofluorescence detection for Ki67 in HuCCT1 cells. Scale bar, 50 µm. (***P* <0.01, as indicated).

**Figure S2**

**A** Representative images (left) and quantification (right) of western blots for p53, p-p53 (Ser15), acetyl-p53 (Lys373) and p21 levels in HuCCT1 cells. **B** Representative immunoblot images (left) and statistical analysis (right) of Cyto-c, Bcl-2, Bax, cleaved-caspase-3, 9 and caspase-3, 9 in HuCCT1 cells. **C** Western blot (left) and quantification (right) of PUMA, Bcl-2, Cyto-c expression in HuCCT1 cells either untreated or pretreated with p53 inhibitor PFT-α (20μM) and following transfected with Adv-GFP or Adv-LASS2-GFP. **D** Detection of EMT markers (snail, slug, N-cadherin, E-cadherin, vimentin) by Western blot in HuCCT1 cells. **E** Western blot analysis of ECM-related proteins (MMP2 and MMP9) in HuCCT1 cells. **F** HuCCT1 cells were pre-treated with PFT-α (20μM) for 24 h, and then transfected with Adv-GFP or Adv-LASS2-GFP. Western blot analysis of p53 downstream EMT targets (slug, N-cadherin, and vimentin) expression in different experimental groups of HuCCT1 cells. (**P* <0.05, ***P* <0.01, ****P* <0.001, as indicated).

**Figure S3**

**A** Bound proteins were separated by SDS-PAGE and visualized by staining with Coomassie blue. **B** Exogenous interactions between LASS2 and MDMX or MDM2 detected by co-IP followed by western blot analysis in HuCCT1 cells. **C**, **D** Overexpression of LASS2 downregulates (**C**) MDM2 or (**D**) MDMX mRNA levels in HuCCT1 cells. **E** Detection of MDM2 and MDMX protein expression levels by Western blot analysis in HuCCT1 cells. **F** Western blotting images and quantification of p53 protein in the nucleus and cytoplasm of HuCCT1 cells. (**P*<0.05, ***P*<0.01, as indicated).
