# Supplementary figures and images for: LASS2 enhances p53 protein stability and nuclear import to suppress liver cancer progression through interaction with MDM2/MDMX
Source: Cell Death Discov. 2023 Nov 14;9:414. doi: 10.1038/s41420-023-01709-2 (PMC10646090; doi:10.1038/s41420-023-01709-2)

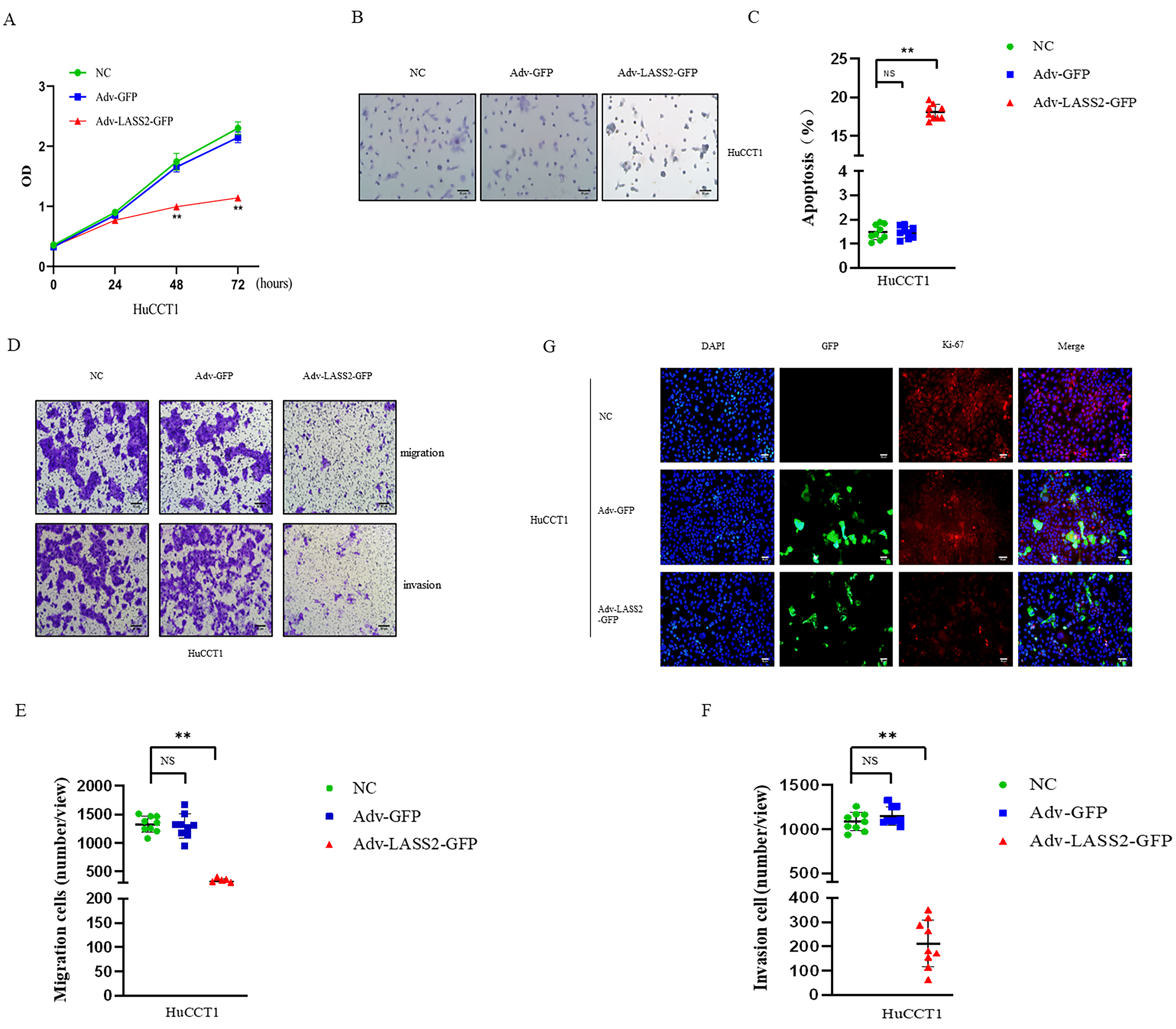

Supplement: Supplementary file 2 — Figure. S1 [file 41420_2023_1709_MOESM2_ESM.tif]

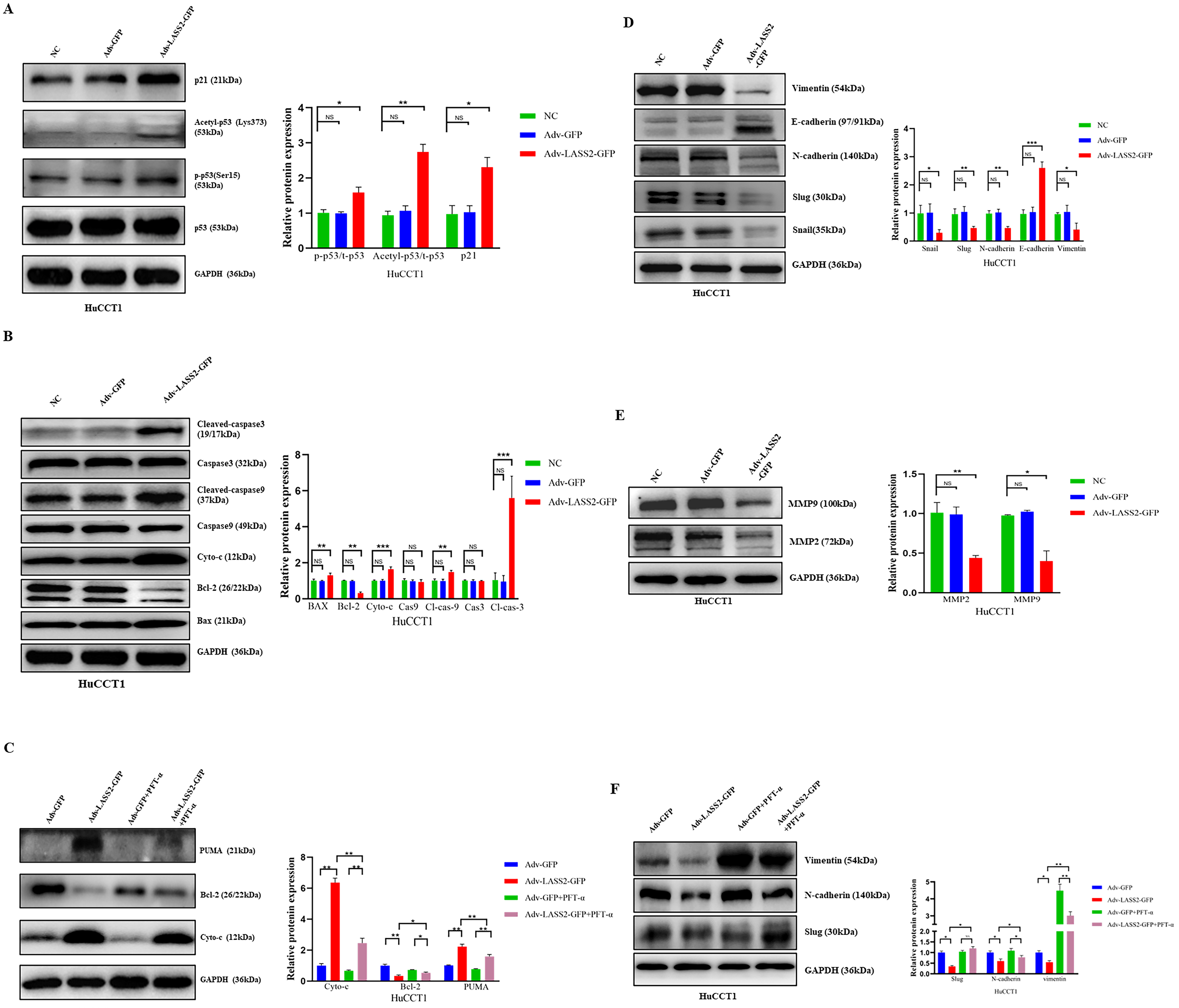

Supplement: Supplementary file 3 — Figure. S2 [file 41420_2023_1709_MOESM3_ESM.tif]

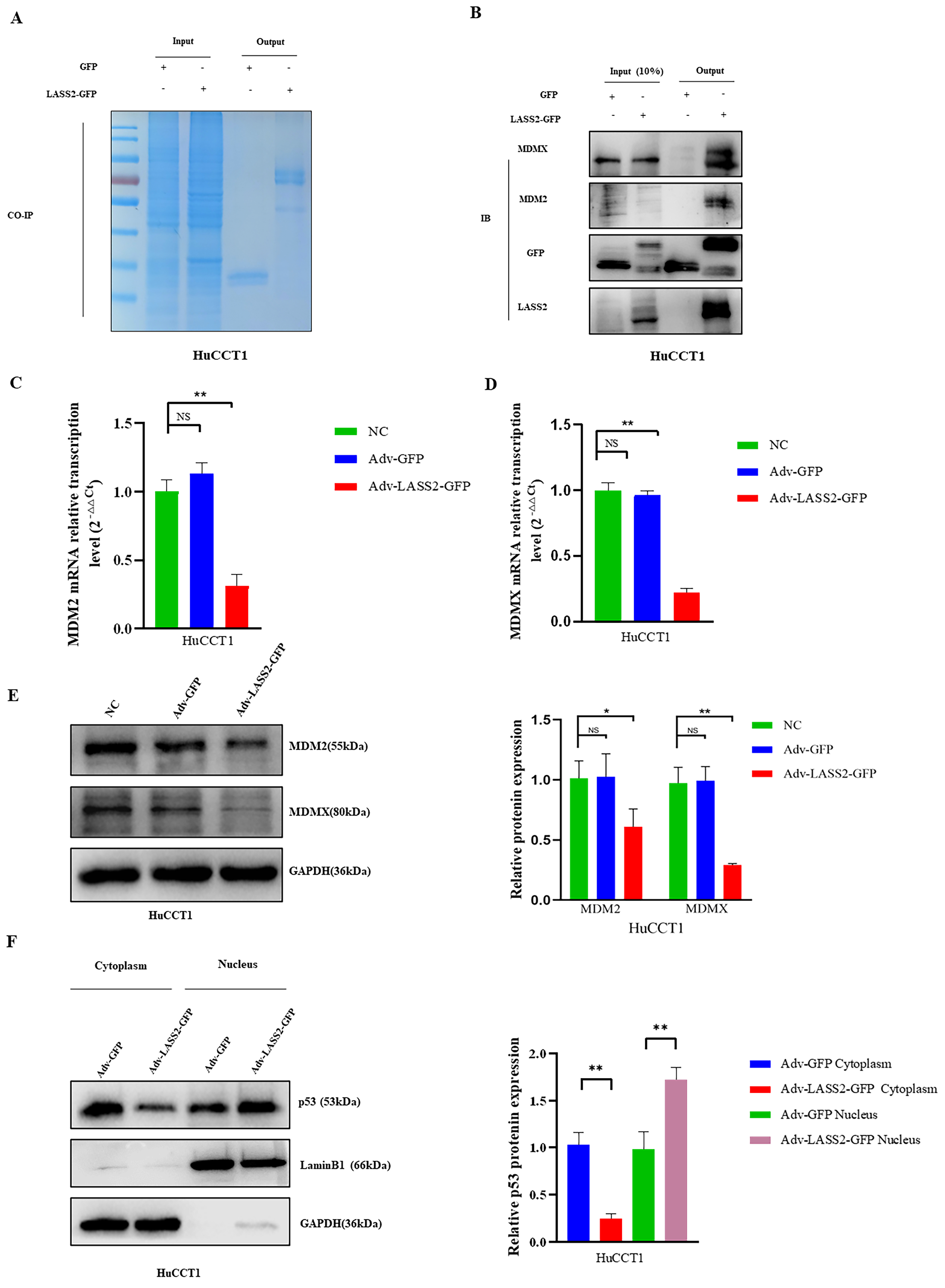

Supplement: Supplementary file 4 — Figure. S3 [file 41420_2023_1709_MOESM4_ESM.tif]
