# Supplementary material for: LASS2 enhances p53 protein stability and nuclear import to suppress liver cancer progression through interaction with MDM2/MDMX
Source: Cell Death Discov. 2023 Nov 14;9:414. doi: 10.1038/s41420-023-01709-2 (PMC10646090; doi:10.1038/s41420-023-01709-2)
Supplement: Supplementary file 5 — Supplemental table 1-5 [file 41420_2023_1709_MOESM5_ESM.docx]

| Protein-protein | Weighted Score |
| --- | --- |
| LASS2-MDM2 | -546.6 |
| LASS2-MDMX | -1327.9 |

Supplemental Table 1.

The protein-protein predicted binding affinity

Supplemental Table 2.

Types of LASS2-MDM2 complex interactions

| Chain A | Residue | Chain B | Residue | Interaction type |
| --- | --- | --- | --- | --- |
| LASS2  LASS2  LASS2  LASS2  LASS2  LASS2  LASS2 | ARG 105 [NH1] | MDM2 | GLU 436 [OE1] | Hbond |
|  | ARG 105 [HE] | MDM2 | GLU 436 [OE1] | Hbond |
|  | GLN 106 [NE2] | MDM2 | LYS 446 [O] | Hbond |
|  | GLN 106 [NE2] | MDM2 | THR 488 [O] | Hbond |
|  | GLN 106 [O] | MDM2 | LYS 446 [H] | Hbond |
|  | ARG 105 [NH1] | MDM2 | GLU 436 [OE1] | Salt bridge |
|  | ARG 105 [NE] | MDM2 | GLU 436 [OE1] | Salt bridge |

Supplemental Table 3.

Types of LASS2-MDMX complex interactions

| Chain A | Residue | Chain B | Residue | Interaction type |
| --- | --- | --- | --- | --- |
| LASS2  LASS2  LASS2  LASS2  LASS2  LASS2 | THR 285 [OG1] | MDMX | ARG 255 [O] | Hbond |
|  | LYS 382 [HZ3] | MDMX | GLU 260 [OE1] | Hbond |
|  | LYS 382 [HZ1] | MDMX | GLU 260 [OE2] | Hbond |
|  | LYS 350 [HZ1] | MDMX | SER 264 [OG] | Hbond |
|  | LYS 396 [HZ3] | MDMX | GLU 280 [OE1] | Hbond |
|  | LYS 396 [HZ1] | MDMX | GLU 280 [OE2] | Hbond |
| LASS2 | LYS 973 [HZ2] | MDMX | GLU 292 [OE1] | Hbond |
| LASS2 | LYS 973 [HZ3] | MDMX | GLU 292 [OE2] | Hbond |
| LASS2 | LYS 404 [HZ1] | MDMX | ARG 303 [O] | Hbond |
| LASS2 | GLN 394 [NE2] | MDMX | SER 310 [O] | Hbond |
| LASS2 | ASN 276 [H] | MDMX | PRO 311 [O] | Hbond |
| LASS2 | TYR 951 [ HH] | MDMX | SER 315 [O] | Hbond |
| LASS2 | LYS 947 [HZ1] | MDMX | THR 316 [ OG] | Hbond |
| LASS2 | CYS 268 [SG] | MDMX | ASP 318 [O] | Hbond |
| LASS2 | LYS 981 [HZ1] | MDMX | ASP 318 [OD2] | Hbond |
| LASS2 | VAL1002 [H] | MDMX | SER 322 [OG] | Hbond |
| LASS2 | LYS 881 [HZ1] | MDMX | SER 326 [OG] | Hbond |
| LASS2 | ARG 459 [NH2] | MDMX | CYS 327 [SG] | Hbond |
| LASS2 | LYS 462 [ HZ3] | MDMX | SER 328 [OG] | Hbond |
| LASS2 | LYS 882[ HZ3] | MDMX | SER 329 [O] | Hbond |
| LASS2 | LYS 882[ HZ3] | MDMX | ASP 330 [O] | Hbond |
| LASS2 | LYS 882 [HZ1] | MDMX | ASP 330 [OD2] | Hbond |
| LASS2 | ASP 212 [OD1] | MDMX | ARG 247 [HE] | Hbond |
| LASS2 | ASP 211 [OD2] | MDMX | ARG247 [NH2] | Hbond |
| LASS2 | SER 210 [OG] | MDMX | ARG 250 [HE] | Hbond |
| LASS2 | GLU 209 [OE2] | MDMX | ARG 250 [NH1] | Hbond |
| LASS2 | GLY 284 [O] | MDMX | ASP 253 [ H] | Hbond |
| LASS2 | GLU 401 [OE2] | MDMX | ARG 305 [H] | Hbond |
| LASS2 | ASP 428 [OD2] | MDMX | ARG 305 [NH1] | Hbond |
| LASS2 | SER 272 [OG] | MDMX | SER 314 [H] | Hbond |
| LASS2 | ASP 270 [OD2] | MDMX | SER 315 [H] | Hbond |
| LASS2 | GLU 263 [OE1] | MDMX | LYS 319 [HZ1] | Hbond |
| LASS2 | GLU 263 [OE2] | MDMX | LYS 319 [HZ3] | Hbond |
| LASS2 | GLY 264 [O] | MDMX | LYS 319 [HZ2] | Hbond |
| LASS2 | SER 265 [O] | MDMX | GLN 323 [NE2] | Hbond |
| LASS2 | LYS 382 [NZ] | MDMX | GLU 260 [OE1] | Salt bridge |
| LASS2 | LYS 382 [NZ] | MDMX | GLU 260 [OE2] | Salt bridge |
| LASS2 | LYS 396 [NZ] | MDMX | GLU 280 [OE1] | Salt bridge |
| LASS2 | LYS 396 [NZ] | MDMX | GLU 280 [OE2] | Salt bridge |
| LASS2 | LYS 973 [NZ] | MDMX | GLU 292 [OE1] | Salt bridge |
| LASS2 | LYS 973 [NZ] | MDMX | GLU 292 [OE2] | Salt bridge |
| LASS2 | HIS 756 [ND1] | MDMX | GLU 317 [OE1] | Salt bridge |
| LASS2 | LYS 981 [NZ] | MDMX | ASP 318 [OD1] | Salt bridge |
| LASS2 | LYS 981 [NZ] | MDMX | ASP 318 [OD2] | Salt bridge |
| LASS2 | LYS 882 [NZ] | MDMX | ASP 330 [OD2] | Salt bridge |
| LASS2 | GLU 223 [OE2] | MDMX | ARG 227 [NE] | Salt bridge |
| LASS2 | GLU 223 [OE1] | MDMX | ARG 227 [NH1] | Salt bridge |
| LASS2 | GLU 223 [OE2] | MDMX | ARG 227 [NH1] | Salt bridge |
| LASS2 | ASP 212 [OD1] | MDMX | ARG 247 [NE] | Salt bridge |
| LASS2 | ASP 211 [OD1] | MDMX | ARG 247 [NH1] | Salt bridge |
| LASS2 | ASP 211 [OD2] | MDMX | ARG 247 [NH1] | Salt bridge |
| LASS2 | ASP 211 [OD1] | MDMX | ARG 247 [NH2] | Salt bridge |
| LASS2 | ASP 211 [OD2] | MDMX | ARG 247 [NH2] | Salt bridge |
| LASS2 | ASP 212 [OD1] | MDMX | ARG 247 [NH2] | Salt bridge |
| LASS2 | ASP 212 [OD2] | MDMX | ARG 247 [NH2] | Salt bridge |
| LASS2 | GLU 209 [OE1] | MDMX | ARG 250 [NH1] | Salt bridge |
| LASS2 | GLU 209 [OE2] | MDMX | ARG 250 [NH1] | Salt bridge |
| LASS2 | GLU 209 [OE1] | MDMX | ARG 250 [NH2] | Salt bridge |
| LASS2 | GLU 209 [OE2] | MDMX | ARG 250 [NH2] | Salt bridge |
| LASS2 | ASP 428 [OD1] | MDMX | ARG 305 [NH1] | Salt bridge |
| LASS2 | ASP 428 [OD2] | MDMX | ARG 305 [NH1] | Salt bridge |
| LASS2 | ASP 428 [OD2] | MDMX | ARG 305 [NH2] | Salt bridge |
| LASS2 | GLU 263 [OE1] | MDMX | LYS 319 [NZ] | Salt bridge |

Supplemental Table 4.

The sequence of primers for qPCR analysis

| Primer | Forward | Reverse |
| --- | --- | --- |
| β-actin | 5′-TCCTGTGGCATCCACGAAACT-3′ | 5′-GAAGCATTTGCGGTGGACGAT-3′ |
| *h*LASS2 | 5′-ATCGTCTTCGCCATTGTT-3′ | 5′-CGGTCACTGCGTTCATCT-3′ |
| *h*MDM2 | 5′-GGGATGAGAGCCGAATAAG-3′ | 5′-TGCAAAAGGCACTGAGATAA-3′ |
| *h*MDMX | 5′-TCTCGCTCTCGCACAGGATCACA-3′ | 5′-AACCACCAAGGCAGGCCAGCTA-3′ |

Supplemental Table 5.

Antibodies, source and dilution

| Antibodies | Source | Dilution |
| --- | --- | --- |
| LASS2 (WB)  GFP | SantaCruz, sc-390745  HuaBio, ET1607-31 | 1:500  1:5000 |
| p53 | Proteintech, China, 60283-2-Ig | 1:5000 |
| Acetyl-p53 (WB) | Abcam, ab62376 | 1:1000 |
| p-p53 | CST, #9284T | 1:1000 |
| Bcl-2 | HuaBio, ET1603-11 | 1:1000 |
| Bax | HuaBio, ET1603-34 | 1:1000 |
| Cyto-c | HuaBio, ET1610-60 | 1:1000 |
| Caspase3 | CST, #14220 | 1:1000 |
| cleaved-caspase 3 | CST, #9664 | 1:1000 |
| caspase 9 | CST, #9505 | 1:1000 |
| cleaved-caspase 9 | CST, #7237 | 1:1000 |
| E-cadherin | Proteintech, 20874-1-AP | 1:2000 |
| N-cadherin | HuaBio, ET1607-37 | 1:2000 |
| Snail | Proteintech, 13099-1-AP | 1:2000 |
| Slug | SantaCruz, sc-166476 | 1:500 |
| Vimentin | HuaBio, ET1610-39 | 1:3000 |
| MMP2 | HuaBio, ET1606-4 | 1:1000 |
| MMP9 | SantaCruz, sc-393859 | 1:5000 |
| LaminB1 | HuaBio, ET1606-27 | 1:5000 |
| MDMX/MDM4 | Proteintech, 17914-1-AP | 1:1000 |
| MDM2 | Proteintech, 66511-1-Ig | 1:1000 |
| Ki-67 | Proteintech, 27309-1-AP | 1:1000 |
| GAPDH | HuaBio, EM1101 | 1:5000 |
| Secondary antibody for WB  (Goat-anti-rabbit) | HuaBio, HA1001 | 1:5000 |
| Secondary antibody for WB  (Goat-anti-mouse) | HuaBio, HA1006 | 1:5000 |
| LASS2 (IHC) | SantaCruz, sc-390745 | 1:100 |
| Acetyl-p53 (IHC) | Abcam, ab62376 | 1:400 |
| Secondary antibody for IHC  (Goat-anti-rabbit) | Servicebio, G1213-100UL | 1:200 |
| Secondary antibody for IHC  (Goat-anti-mouse) | Servicebio, G1214-100UL | 1:200 |
